# Supplementary material for: Cognitive Job Demands and Sports Participation among Young Workers: What Moderates the Relationship?
Source: Int J Environ Res Public Health. 2024 Jan 28;21(2):144. doi: 10.3390/ijerph21020144 (PMC10888168; doi:10.3390/ijerph21020144)
Supplement: Supplementary file 1 [file ijerph-21-00144-s001.zip › ijerph-2727583-supplementary.pdf]

SUPPLEMENTALMATERIALS

A. Information operationalisations

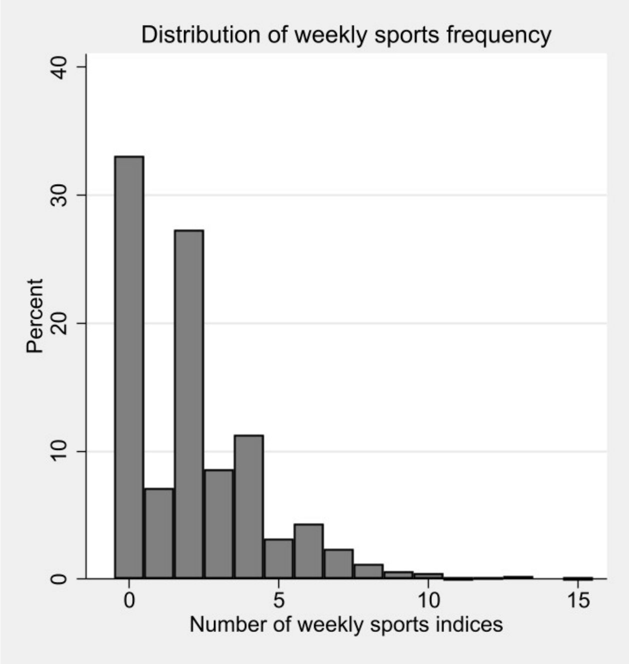

Figure S1. Histogram of weekly sports participation.

Table S1. Overview of educational levels and corresponding ISCED levels.

Level	ISCED	Years of education
(unfinished) Primary school	1	6
LBO/VBO/VMBOpraktijk/MBO1	2	8
MAVO/VMBO theorie	2	10.5
MBO2/MBO3/MBO4	3	10.5
HAVO/VWO/HBS/propedeuse	3	11.5
HBO or WO bachelor	5/6	15
HBO or WO master/postdoctoraal	7	16.5

Note: see for overview https://eurydice.eacea.ec.europa.eu/national-education-systems/netherlands/overview.

B. Correlationmatrix

Table S2. Correlation matrix.

	1	2	3	4	5	6	7	8	9	10	11
Weekly sports participation - no indices	−0.036	−0.238 *	0.096 *	−0.156 *	−0.005	0.094 *	0.037	0.058 *	−0.037	0.026	0.160 *
Weekly sports participation - 1 to 3 indices	−0.013	0.167 *	0.000	0.081 *	−0.043	−0.042	0.013	0.039	−0.045 *	−0.022	−0.089 *
Weekly sports participation – more than 3 indices	0.055 *	0.085 *	−0.110 *	0.088 *	0.054 *	−0.061 *	−0.057 *	−0.111 *	0.092 *	−0.004	−0.083 *
1 Cognitive job demands	1.000										
2 Years of education (6 years = 0)	0.137 *	1.000									
3 Being a parent (= 1)	−0.051 *	−0.043	1.000								
4 Control over work time and work location	0.006	0.383 *	−0.013	1.000							
5 Male (= 1)	−0.072 *	−0.023	−0.035	0.111 *	1.000						
6 Age (18 years old = 0)	0.017	−0.019	0.394 *	0.045 *	0.075 *	1.000					
7 Hampered by health (= 1)	0.126 *	−0.085 *	0.000	−0.043	−0.069 *	−0.037	1.000				
8 Cohabiting with a partner (= 1)	0.023	0.023	0.446 *	−0.006	−0.075 *	0.198 *	0.005	1.000			
9 Working hours (20 hours = 0)	0.210 *	0.093 *	−0.206 *	0.098 *	0.318 *	0.016	−0.060 *	−0.092 *	1.000		
10 Permanent contract (= 1)	0.072 *	−0.066 *	0.116 *	−0.007	0.046 *	0.213 *	−0.003	0.092 *	0.037	1.000	
11Physical labour	−0.028	−0.430 *	0.037	−0.580 *	−0.074 *	−0.047 *	0.055 *	0.009	−0.064 *	0.001	1.000

Source: Transition into active living, 2021, 2022; n = 2032. * P < 0.05 (two-tailed).

C. Tables additional analyses

Table S3. Outcomes from OLS regression estimating weekly sports frequency.

	Model1			Model2			Model3			Model4		
	b		SE	b		SE	b		SE	b		SE
Cognitive job demands	0.103		0.092	0.292		0.327	0.282	**	0.106	0.079		0.153
Years of education	0.115	***	0.022	0.144	**	0.053	0.115	***	0.022	0.115	***	0.022
Parent (no = ref.)	−0.257	*	0.119	−0.255	*	0.119	0.669	*	0.295	−0.258	*	0.119
Control over work time and work location	0.089	+	0.052	0.086		0.053	0.095		0.052	0.068		0.123
Cognitive job demands*												
Years of education				−0.022		0.037						
Parent (no = ref.)							−0.685	***	0.200			
Control over work time and work location										0.016		0.083
Intercept	1.052	***	0.295	0.816	+	0.490	0.823	**	0.301	1.078	***	0.325

Source: Transition into active living, 2021, 2022; n = 2032. * P < 0.05; ** P < 0.01, *** P < 0.001 (two-tailed)

Note: All models include the control variables from Table 2 Model 3.

Table S4. Outcomes from multinomial logistic regression estimating sports participation differentiated by sex with base outcome 'no weekly sports indices'.

	**Female(n=1267)**									**Male(n=765)**								
	Model1			Model2			Model3			Model1			Model2			Model3		
	b		SE	b		SE	b		SE	b		SE	b		SE	b		SE
**One to three weekly sports indices**																		
Cognitive job demands	−0.079		0.136	1.262	*	0.529	−0.207		0.162	−0.034		0.190	0.488		0.689	0.109		0.221
Years of education	0.132	***	0.033	0.337	***	0.085	0.129	***	0.033	0.257	***	0.046	0.335	**	0.112	0.255	***	0.046
Parent (no = ref.)	−0.243		0.180	−0.227		0.181	−0.863	*	0.440	−0.027		0.234	−0.030		0.234	0.635		0.587
Control over work time and work location	0.153	+	0.078	0.153	+	0.079	0.152	+	0.079	0.043		0.103	0.034		0.104	0.053		0.104
Cognitive job demands*																		
Years of education				−0.154	**	0.058							−0.060		0.078			
Parent (no = ref.)							0.439		0.285							−0.503		0.411
Intercept	−0.805	+	0.430	−2.509	**	0.791	−0.638		0.445	−1.367	*	0.606	−2.012	+	1.027	−1.540	*	0.621
**More than three weekly sports indices**																		
Cognitive job demands	−0.029		0.163	0.973		0.642	0.028		0.185	0.367	+	0.199	0.352		0.696	0.586	**	0.225
Years of education	0.124	**	0.040	0.280	**	0.103	0.127	**	0.040	0.129	**	0.047	0.128		0.118	0.125	**	0.047
Parent (no = ref.)	−0.492	*	0.223	−0.477	*	0.224	0.007		0.548	−0.157		0.270	−0.156		0.270	1.196	+	0.680
Control over work time and work location	0.189	*	0.093	0.190	*	0.093	0.187	*	0.093	0.130		0.113	0.129		0.114	0.149		0.114
Cognitive job demands*																		
Years of education				−0.117	+	0.070							0.000		0.080			
Parent (no = ref.)							−0.366		0.372							−1.017	*	0.477
Intercept	−1.799	***	0.528	−3.070	***	0.966	−1.880	***	0.541	−1.152	+	0.638	−1.118		1.071	−1.453	*	0.656

Source: Transition into active living, 2021, 2022; n = 2032. + P < 0.10; * P < 0.05; ** P < 0.01; *** P < 0.001 (two-tailed)

Note: All models include the control variables from Table 2 Model 3.
